# Subclinical Myocardial Dysfunction Assessment Using Speckle Tracking Echocardiography in Patients With Psoriasis: A Pilot Meta‐Analysis

**DOI:** 10.1002/clc.70047

**Published:** 2024-12-11

**Authors:** Hritvik Jain, Jyoti Jain, Debankur Dey, Rishika Modi, Omar Alomari, Mushood Ahmed, Jagjot Singh, Ramez M. Odat, Raheel Ahmed, Abdulqadir J. Nashwan

**Affiliations:** ^1^ Department of Internal Medicine All India Institute of Medical Sciences (AIIMS) Jodhpur India; ^2^ Department of Internal Medicine Medical College and Hospital Kolkata India; ^3^ Department of Internal Medicine Government Medical College Nagpur India; ^4^ Department of Internal Medicine Hamidiye International Faculty of Medicine, University of Health Sciences Istanbul Turkey; ^5^ Department of Internal Medicine Rawalpindi Medical University Rawalpindi Pakistan; ^6^ Department of Internal Medicine Government Medical College Amritsar India; ^7^ Department of Internal Medicine Faculty of Medicine, Jordan University of Science and Technology Irbid Jordan; ^8^ Department of Cardiology National Heart and Lung Institute, Imperial College London London UK; ^9^ Department of Public Health Hamad Medical Corporation Doha Qatar; ^10^ Department of Public Health College of Health Sciences, QU Health, Qatar University Doha Qatar

**Keywords:** cardiac strain, cardiovascular disease, echocardiography, psoriasis

## Abstract

**Introduction:**

Psoriasis is a systemic inflammatory disease associated with elevated cardiovascular risk due to inflammatory and oxidative stress. Two‐dimensional speckle‐tracking echocardiography (2D‐STE) can detect both regional and global myocardial strain. Impairment of ventricular strain can assist in the early detection of myocardial dysfunction. Subclinical myocardial dysfunction in psoriasis has not yet been elucidated with inconsistent results.

**Methods:**

A systematic literature search of various databases was conducted to identify studies comparing global longitudinal strain (GLS) and global circumferential strain (GCS) between patients with psoriasis and healthy controls. Standardized mean differences (SMD) with 95% confidence intervals (CI) were pooled using the inverse‐variance random‐effects model in Review Manager Software Version 5.4.1.

**Results:**

Eleven studies with 879 participants (501 patients with psoriasis and 378 healthy controls) were included. Psoriasis was associated with a statistically significant reduction in GLS [SMD: –1.04; 95% CI: –1.45, –0.62; *p* < 0.00001] and GCS [SMD: –0.66; 95% CI: –1.27, –0.05; *p* = 0.03] compared to healthy controls.

**Conclusion:**

This study demonstrated that patients with psoriasis are at an elevated risk of subclinical myocardial dysfunction, as shown by the reduced GLS and GCS. Early assessment of subclinical impairment in psoriasis will allow targeted intervention and may mitigate future adverse cardiovascular events. Prospective studies with larger sample sizes are warranted to validate these results.

## Graphical abstract

The graphical abstract illustrates the meta‐analysis findings of significantly reduced global longitudinal strain and global circumferential strain in patients with psoriasis compared to healthy controls.

**Abbreviations**
2D‐STE: 2‐dimensional speckle‐tracking echocardiography
CVD: cardiovascular disease
EF: ejection fraction
GCS: global circumferential strain
GLS: global longitudinal strain
LV: left ventricle
LVEF: left ventricular ejection fraction

## 1 Introduction

Psoriasis is a long‐term inflammatory skin condition characterized by gray scaly patches typically found on the extensor surfaces of the body [1]. Psoriasis has no definitive cure, and waxes and wanes as it progresses [2]. According to the Global Burden of Disease 2019 study, the prevalence rate is approximately 2%–3%, reaching a maximum of 11% in some Northern European countries [3]. Psoriasis is associated with numerous clinical conditions including psoriatic arthritis, uveitis, psychiatric conditions, and Crohn's disease [4]. Psoriasis is also associated with elevated cardiovascular risk [5, 6, 7]. This has been attributed to inflammation and oxidative stress caused by psoriasis, along with an increased risk of mortality [8, 9, 10].

The pathophysiological mechanism behind the systemic inflammatory state in psoriasis involves T helper 1 and T helper 17 lymphocytes, which leads to endothelial damage and premature atherosclerotic progression [11, 12, 13, 14, 15, 16]. Subclinical myocardial injury contributes to increased cardiovascular mortality [17]. Two‐dimensional speckle tracking echocardiography (2D‐STE) is a valid method for evaluating ventricular dysfunction by measuring myocardial deformation (strain) and providing an objective assessment of the regional left ventricular function [18, 19, 20]. 2D‐STE is a non‐Doppler ultrasound that is based on frame tracking of minute echo‐dense speckles noted within the myocardium and subsequent myocardial motion measurement. 2D‐STE quantitatively measures myocardial tissue velocity, strain, and strain rate. However, subclinical myocardial dysfunction in psoriasis has not yet been studied. Hence, we conducted a pilot systematic review and meta‐analysis of the available literature to provide evidence for this association.

## 2 Materials and Methods

This systematic review and meta‐analysis was designed, conducted, and reported according to the guidelines of the Preferred Reporting Items for Systematic Review and Meta‐Analysis (PRISMA) 2020 [Supporting Information S1: 1] [21]. The review protocol was prospectively registered in the PROSPERO Registry (CRD42024567925).

### 2.1 Search Strategy and Study Selection

Two reviewers (J.J. and D.D) systematically searched the major bibliographic databases, including Medline (via PubMed), Embase, Google Scholar, Scopus, Cochrane Library, and clinicaltrials.gov, from their inception until June 2024. The search strategy consisted of keywords, such as “psoriasis,” “echocardiography,” “two‐dimensional speckle tracking,” and “strain.” Boolean operators (AND and OR) were combined with these keywords to create database‐specific search strategies (Supporting Information S1: Table 2). Studies that adhered to the following inclusion criteria were considered: (i) randomized controlled trials or observational studies (case‐control, cross‐sectional, or cohort); (ii) patients with psoriasis in one arm; (iii) healthy controls in the other arm; (iv) all study participants underwent echocardiography with subsequent myocardial strain analysis; and (v) reported the outcomes of interest. The exclusion criteria were as follows: (i) publications that were reviewed, case reports, case series, letters to the editor, viewpoints, or correspondence; (ii) no healthy controls as comparators; and (iii) did not perform echocardiography. No restrictions were imposed on publication year or language. Manual screening of the reference lists was conducted to identify additional studies.

Before the initiation of the screening process, duplicate publications were removed using the EndNote X7 Software (Clarivate Analytics, USA). Two reviewers (J.J. and D.D.) independently screened the titles and abstracts for inclusion and excluded articles that did not meet the inclusion criteria. Subsequently, the full text of the shortlisted articles was retrieved and thoroughly evaluated. Any disagreements between the reviewers were resolved by reaching a consensus or consulting a third reviewer (H.J.). All the included studies reported that patients with a history of any CVDs were excluded from the analysis. However, no study mentioned any detail about the tests or tools used.

### 2.2 Data Extraction and Quality Assessment

Data extraction was carefully performed by three reviewers (H.J., D.D., and O.A.). The following data were extracted from each study: author's name, publication year, study design, country, echocardiographic measurement with strain analysis software, number of participants, disease duration, age group, male sex (%), body mass index, smoking (%), and outcomes of interest. Quality assessment of all included studies was performed using the Newcastle–Ottawa Scale [22]. Two reviewers (D.D. and O.A.) independently conducted this assessment and cross‐checked each other's assessments.

### 2.3 Data Synthesis

Statistical analysis for this meta‐analysis was conducted using the Review Manager Version 5.4.1 (RevMan) software (Nordic Cochrane Collaboration, Copenhagen, Denmark). For continuous outcomes, standardized mean differences (SMD) with 95% confidence intervals (CI) were pooled using an inverse‐variance random‐effects model. For the assessment of statistical heterogeneity, the Higgins I^2^ metric was used with < 50% denoting “low,” 50%–75% denoting “moderate,” and > 75% denoting “high” heterogeneity [23]. To address high statistical heterogeneity, a leave‐one‐out sensitivity analysis was conducted by serially omitting each study in the pooled estimate to identify the study contributing the most to the high heterogeneity. Statistical significance was considered if: (i) the 95% CI was not crossing the digit “1,” and (ii) the *p*‐value was < 0.05. Funnel plots were visualized to evaluate publication bias.

## 3 Results

**Figure 1:**
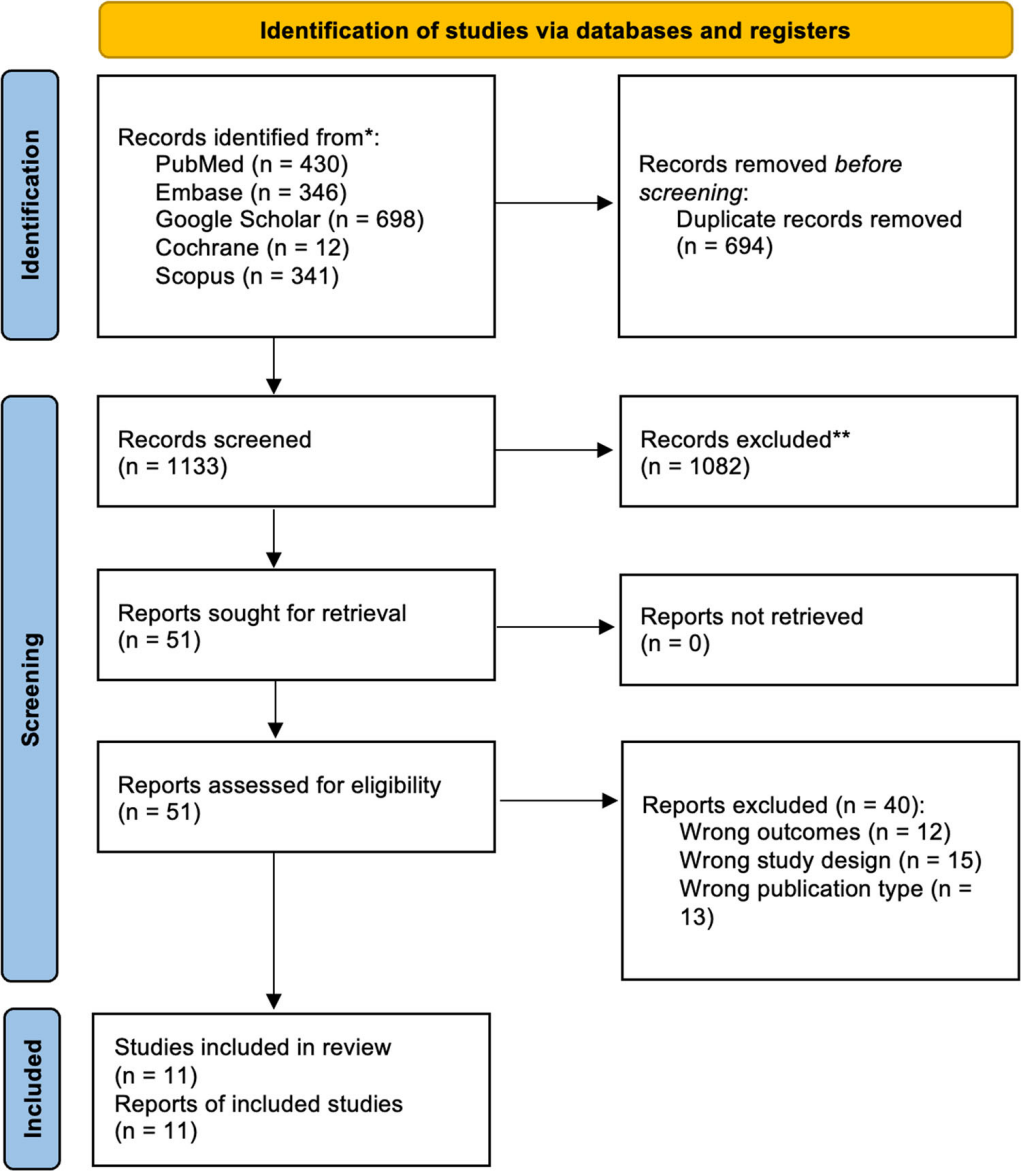
The preferred reporting items for systematic reviews and meta‐analyses (PRISMA) 2020 flowchart.

A total of 1826 potentially relevant records were identified using various databases. After removing duplicates (*n* = 694), 1133 articles were subjected to preliminary screening based on titles and abstracts. A total of 1082 articles were excluded. Subsequently, 51 articles were subjected to a full‐text assessment based on the inclusion criteria. Of these 51, 40 were excluded for various reasons, including wrong outcomes (*n* = 12), wrong study design (*n* = 15), and wrong publication type (*n* = 13). Ultimately, 11 studies were included in this meta‐analysis [19, 24, 25, 26, 27, 28, 29, 30, 31, 32, 33]. The selection process is shown in the PRISMA flowchart (Figure 1). The quality of this systematic review was evaluated using the AMSTAR‐2 (A Measurement Tool to Assess Systematic Review) tool (Supporting Information S1: 2).

### 3.1 Baseline Study Data and Quality Assessment

**Figure 2:**
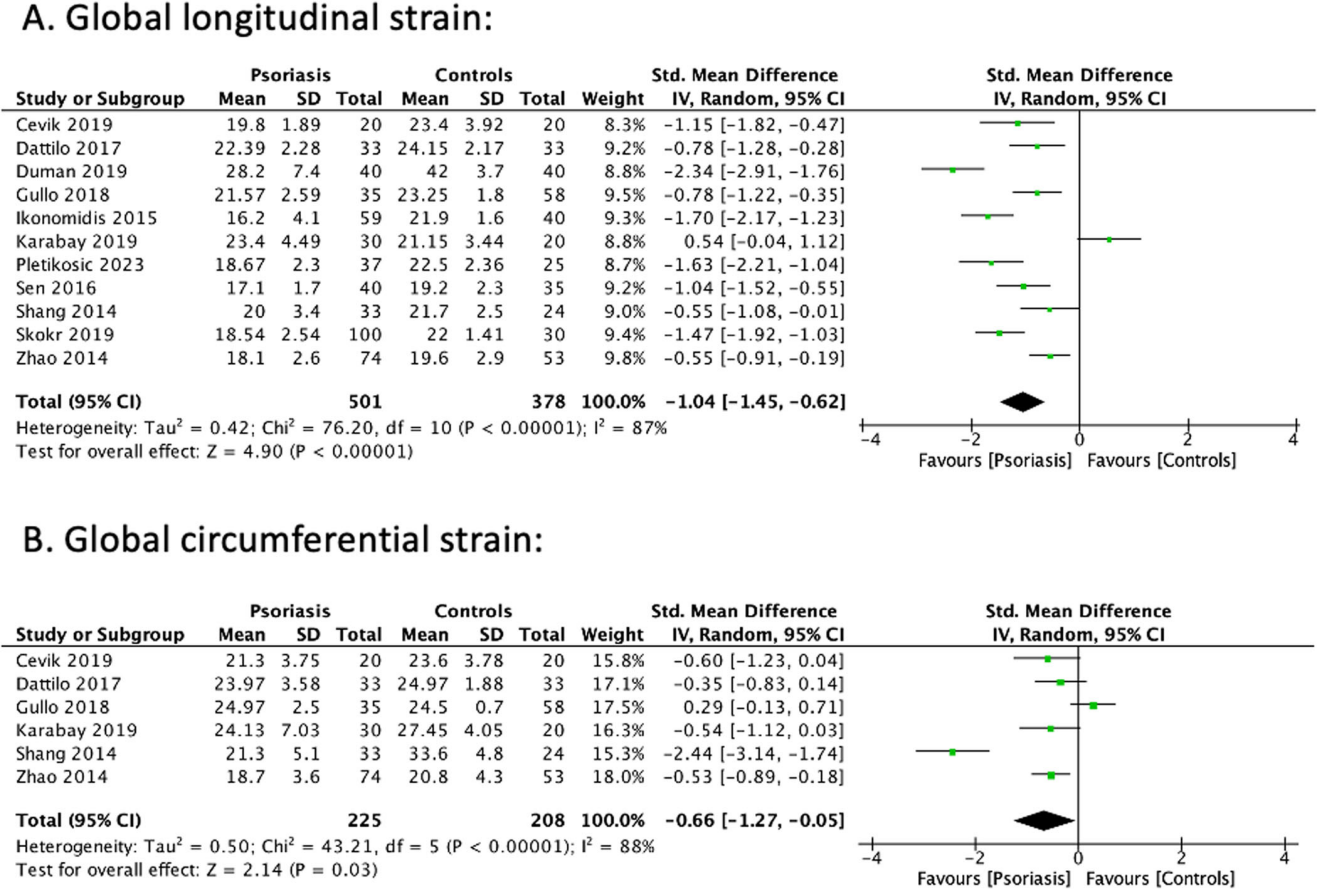
Individual and pooled analyses comparing patients with psoriasis and healthy controls. (A) Global longitudinal strain and (B) Global circumferential strain.

Eleven of the included studies provided data on 879 participants (501 psoriasis patients and 378 healthy controls). All studies had a case‐control design and were published between 2014 and 2023. The detailed baseline characteristics of the included studies are presented in Table 1. The inclusion and exclusion criteria for the studies are presented in Supporting Information S1: Table 3. All studies were deemed of “high” quality according to the Newcastle‐Ottawa Scale (Supporting Information S1: Table 4). Low and moderate risk of publication bias was noted for GLS and GCS respectively (Supporting Information S1: Figures 1 and 2).

**Table 1:** Baseline characteristics of included studies.

Author (Year)	Country	Study design	Echocardiographic measurement with strain analysis software	Number of participants		Disease duration; [Mean ± SD; Median (IQR)]	Age; [mean ± SD or median (IQR)]		Males (%)		BMI; mean ± SD		Smoking (%)	
				Ps	C		Ps	C	Ps	C	Ps	C	Ps	C
Cevik 2019	Turkey	Case control	Philips IE33‐model echocardiography machine (Phillips Medical Systems, Andover, Maryland, USA).	20	20	8.7 (5–14) months	14.2 ± 0.89	14.05 ± 0.88	40	40	NR	NR	NR	NR
Dattilo 2017	Italy	Case control	MyLab Alpha, Esaote, Florence, Italy for echocardiography.XStrain; Esaote for strain analysis.	33	33	5.6 ± 1.7 years	35.6 ± 5.7	36.3 ± 5.9	60.6	54.5	24 ± 3	23.2 ± 2.4	0	0
Duman [26]	Turkey	Case control	Philips iE33 Medical ultrasound Systems device for echocardiography.QLAB, Philips for strain analysis.	40	40	7–8 years	36 (20‐60)	37 (20‐54)	42.5	47.5	24.7 ± 7.7	23.3 ± 4.4	52.5	55
Gullo [19]	Italy	Case control	VIVID‐7 ultrasound machine (GE Vingmed Ultrasound) for echocardiography.EchoPAC, version 8.0.0 for strain analysis.	35	58	Newly diagnosed	45 (23 – 59)	45 (24 – 66)	25.7	39.6	24.7 ± 2.61	24.79 ± 2.73	0	0
Ikonomidis [27]	Greece	Case control	Vivid 7 (GE Medical Systems, Milwaukee, USA) for echocardiography.EchoPAC for strain analysis.	59	40	5.1 ± 1.25 years	51 ± 12.2	49 ± 13	63	55	NR	NR	47	45
Karabay [28]	Turkey	Case control	Philips iE33 ultrasound system (Konin‐klijke Philips Electronics N.V., Amsterdam, Netherlands)	30	20	9.6 years (mean)	34.63 ± 10.2	33.1 ± 7.22	60	50	23.1 ± 1.79	22.6 ± 2.07	0	0
Pletikosic [29]	Croatia	Case control	Vivid 9 (GE Medical System, Milwaukee, USA) for echocardiography.EchoPac (GE Medical Systems, EchoPac PC, version 112) for strain analysis	37	25	12 (6–20.5) years	53 ± 10	42.17 ± 15.72	43.2	NR	25.7 (23.9 – 27.7)	23.4 (22.1 – 24.3)	NR	NR
Sen [34]	Turkey	Case control	GE Vivid 7 system (GE Vingmed Ultrasound AS, Horten, Norway) for echocardiography.EchoPAC PC; GE Vingmed Ultrasound AS for strain analysis.	40	35	12.1 ± 7.2 years	41.1 ± 3.8	41.6 ± 4	37.5	40	25.3 ± 4	25.1 ± 4.8	22.5	20
Shang [30]	China	Case control	Vivid 7 systems (GE Medical systems) for echocardiography.EchoPAC‐PC SW‐only, Version 6.0.0, Vingmed‐GE for strain analysis.	33	24	8.2 years (median)	43.9 ± 12.8	46.6 ± 8.9	45.4	45.8	NR	NR	NR	NR
Skokr [31]	Egypt	Case control	NR	100	30	12.9 ± 4.14 years	30.97 ± 5.99	29.30 ± 7.84	50	50	24.05 ± 3.25	23.37 ± 2.28	NR	NR
Zhao [32]	China	Case control	Vingmed Vivid 7, General, Electric Vingmed Ultrasound (Milwaukee, WI, USA) for echocardiography.EchoPac Version 108.1.5 (General Electric – Vingmed, Horten, Norway) for strain analysis.	74	53	15.7 ± 7 years	47 ± 9	45 ± 9	70	60	25.8 ± 5.0	23.2 ± 2.7	31	11

Abbreviations: BMI, Body mass index; C, Controls; NR, Not reported; Ps, Psoriasis.

### 3.2 Outcomes

#### 3.2.1 Global Longitudinal Strain (GLS)

Data on GLS was reported in all the included studies [19, 24, 25, 26, 27, 28, 29, 30, 31, 32, 33]. The pooled analysis demonstrated that psoriasis was associated with a statistically significant reduction in GLS [SMD: –1.04; 95% CI: –1.45, ‐0.62; *p* < 0.00001] compared to healthy controls (Figure 2A). On sensitivity analysis to address the high statistical heterogeneity (*I* ^2^ = 87%), I^2^ dropped to 81% when Karabay 2019 was omitted [28].

#### 3.2.2 Global Circumferential Strain (GCS)

**Figure 3:**
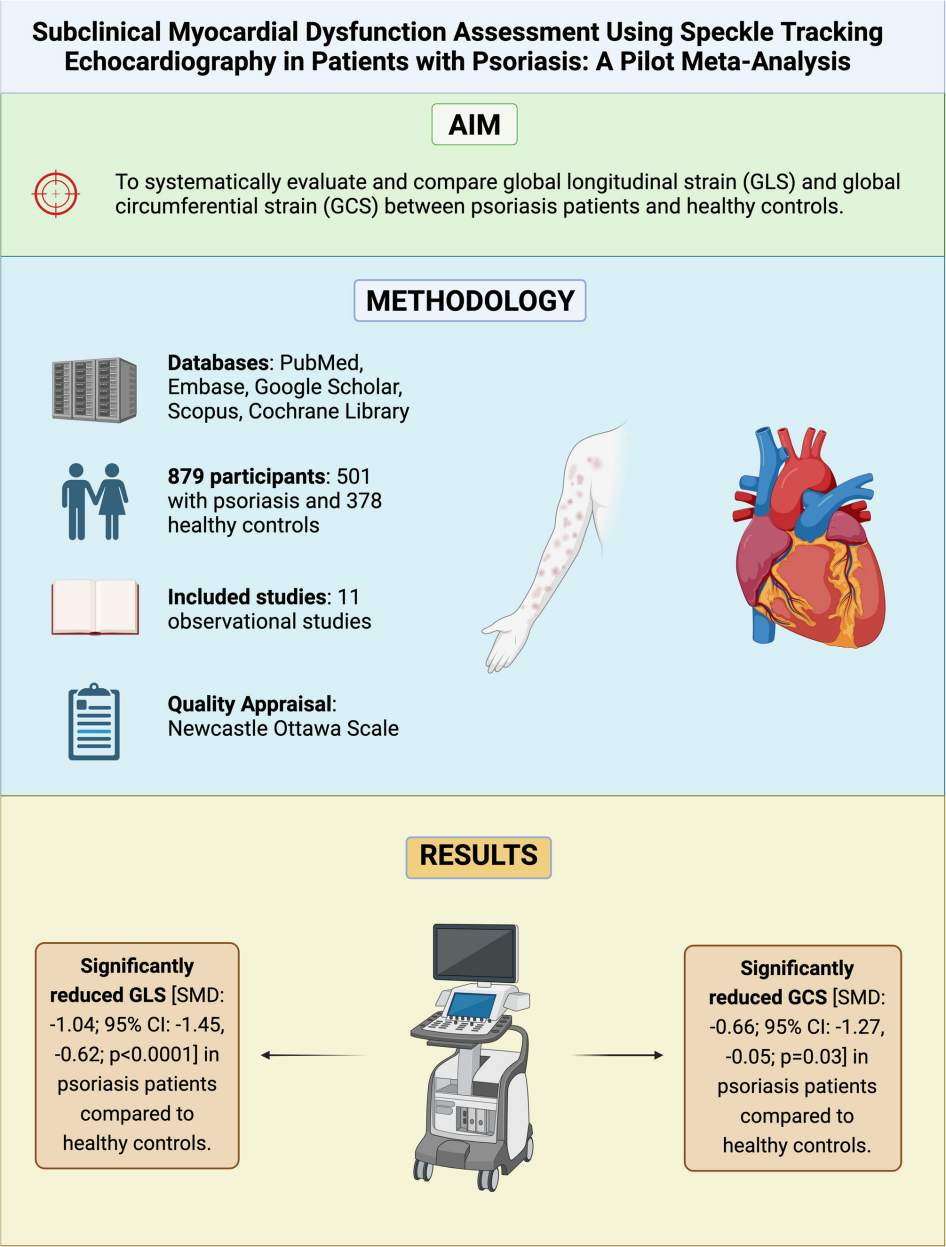
Central illustration summarizing the findings of our meta‐analysis.

Data on GCS was reported in six studies [19, 24, 25, 28, 30, 32]. Pooled analysis demonstrated that psoriasis was associated with a statistically significant reduction in GCS [SMD: –0.66; 95% CI: –1.27, –0.05; *p* = 0.03] compared to healthy controls (Figure 2B). On sensitivity analysis for high heterogeneity (*I* ^2^ = 88%), the *I* ^2^ value dropped to 62% after removing Shang 2014 [30]. The results of the meta‐analysis are summarized in the central illustration Figure 3.

## 4 Discussion

To our knowledge, this is the first meta‐analysis to evaluate factors associated with subclinical myocardial dysfunction in patients with psoriasis. Our study pooled the results from 11 observational studies, encompassing 501 patients with psoriasis and 378 healthy controls. A significant decrease was noted in both GLS [SMD: –1.04; 95% CI: –1.45, –0.62; *p* < 0.00001] and GCS [SMD: –0.66; 95% CI: –1.27, –0.05; *p* = 0.03]. Therefore, our study supports evidence of subclinical myocardial dysfunction in patients with psoriasis.

The association between psoriasis and cardiovascular disease (CVD) has been scrutinized because of the high prevalence of CVDs in these patients, similar to other systemic inflammatory diseases, which cannot be explained by traditional risk factors alone [35, 36]. The role of psoriasis as an independent risk factor for CVD, in the absence of other evident CVD risk factors, remains unclear [37, 38]. There is also growing interest in whether this association extends to subclinical CVD, and exploring this could guide discussions on the early detection and treatment of subclinical CVD in this high‐risk group [39]. Although numerous techniques for cardiovascular risk assessment and management have been proposed, most have focused on endothelial dysfunction and atherosclerosis, with insufficient attention given to myocardial functionality [40, 41]. It was previously believed that psoriasis does not impair left ventricular (LV) systolic function, although several recent studies have changed this understanding.

The assessment of LV function has traditionally involved estimating the ejection fraction (EF) and tissue Doppler imaging (TDI) [42]. EF is the most widely used index of LV function, but its visual component makes the assessment of endocardial excursion subjective, with high interobserver variability [43]. 2D‐STE is a relatively angle‐independent technology that provides a more objective measurement of LV function, making it superior to conventional deformation imaging methods [44]. It is noninvasive and is based on frame‐to‐frame tracking of ultrasonic speckles on greyscale 2D images, detecting minimal abnormalities in systolic and regional LV function [20]. Its clinical utility in detecting early myocardial impairments before changes in LVEF has been demonstrated in other systemic diseases [45, 46, 47, 48]. The use of STE to detect subclinical myocardial dysfunction in patients with rheumatic diseases has been proposed to improve patient stratification and risk management. Similarly, the evaluation of psoriasis using STE showed alterations in the longitudinal and circumferential strains, which is consistent with our results. Reductions in strain and strain rate precede the overall EF reduction, particularly with thickened myocardial walls or small ventricular cavities [49]. This is particularly significant considering that conventionally measured parameters of myocardial function such as LV dimensions, wall thickness, and EF were found to be comparable between patients with psoriasis and healthy controls, reinforcing the reliability of this technique over conventional imaging for the prognostication of cardiac diseases [50, 51, 52]. The mechanism of myocardial dysfunction in psoriasis, as in other systemic inflammatory diseases, has been proposed to be inflammation, although Zhao et al. did not find any correlation between hs‐CRP and 2D STE‐derived strains [32, 53]. This finding suggests that systemic inflammation is not related to myocardial dysfunction in patients with severe psoriasis. Further studies are required to clarify the role of inflammation in myocardial dysfunctions.

A small difference in the extent to which GLS and GCS were affected was noted, with the former being more affected in psoriatic patients, which is not yet fully understood [54, 55]. This can be partly explained by typical myocardial tissue architecture. The myocardium consists of inner oblique, middle, and outer oblique layers created by a single helically folded myocardial muscle band. During systole, the ventricles move inward and the wall thickens; the base moves toward the apex, and the ventricles shorten in the long axis; the apex rotates counterclockwise, and the base rotates clockwise [56]. The LV myocardial fiber architecture changes from oblique in the subepicardium to circumferential in the middle and longitudinal in the subendocardium. The longitudinal function is primarily driven by the deformation of the subendocardial fibers, which are the most vulnerable and sensitive to perfusion changes [34, 57, 58]. In the early stages of heart failure, LV longitudinal function is reduced, while a circumferential strain is preserved because the subendocardial longitudinal fibers are primarily affected [59]. As the disease progresses, macrovascular and microvascular abnormalities and interstitial fibrosis involve the entire ventricle and LV rotation and circumferential deformation weaken, impairing global myocardial function [60]. Therefore, changes in circumferential strain have been proposed as a late response in advanced disease after an initial compensatory increase to preserve EF. One study showed that the change in longitudinal strain was most prominent during the pre‐ejection and ejection phases [61]. Another reason might be the parallel orientation of the ultrasound probe of STE, which helps achieve better resolution in the longitudinal direction, whereas GCS is calculated from the short axis and is thus inevitably affected by the low resolution in the basal and apex layers. Therefore, longitudinal strain has been shown to outperform circumferential strain with higher sensitivity for detecting early myocardial dysfunction [62]. Advances in algorithms and methodologies may improve GCS accuracy in the future. Both GLS and GCS showed high heterogeneity in our study, primarily due to variability in disease severity. Furthermore, only a few studies included patients without known and clinically evident CVD risk factors [24, 25, 28].

Speckle tracking echocardiography (STE), as an advanced imaging modality, can detect changes in myocardial deformation in the subclinical stage. 3D‐STE is considered a validated method for left atrial and left ventricular assessment as compared to two‐dimensional‐speckle tracking echocardiography (2D‐STE). Subclinical LV myocardial involvement can be detected with STE in many CVDs, especially in atherosclerotic/non‐atherosclerotic coronary artery diseases, besides psoriasis [63, 64, 65, 66].

### 4.1 Limitations and Future Recommendations

The results of this study were limited by several factors. First, all studies were observational and, thus, subject to bias in the recruitment process. Data from randomized studies can eliminate these inherent biases and provide comprehensive insights. Second, the process of STE involves a substantial learning curve, and accurate reporting relies on multiple technical considerations, such as the optimization of image acquisition parameters [49, 67]. Future advancements in software performance will help shorten analysis time and enable automated report generation. Third, significant heterogeneity was noted in the assessment of both strain outcomes, despite performing a sensitivity analysis. Comprehensive subgroup analyses based on factors such as disease severity, chamber involvement, or age group could not be performed. Moreover, due to the limited availability of data, we could not assess the difference in the values of other cardiac strains such as Global Radial Strain and Global Area Strain. It is important to mention that left atrial strain analysis could provide additional insights into atrial‐ventricular interactions. Future studies should provide adequate sample sizes to allow exploration of such subgroup differences.

## 5 Conclusion

Psoriasis is associated with subclinical myocardial dysfunction, which can be assessed by abnormal reductions in 2D‐STE parameters, such as GLS and GCS. These reductions in myocardial strain were evident even before the onset of overt CVD in patients with psoriasis. Widespread use of 2D‐STE in patients with psoriasis can detect subtle cardiovascular changes, allowing for early intervention and targeted treatment for this population. Early treatment leads to a reduction in future adverse cardiovascular events, allowing for improvement in mortality rates. Future randomized studies are needed, preferably with larger sample sizes, to corroborate the results of this meta‐analysis.

## Ethics Statement

No ethical approval was required for the study.

## Consent

The authors have nothing to report.

## Conflicts of Interest

The authors declare no conflicts of interest.

## Supporting information

Supporting information.

Supporting information.

## Data Availability

Data sharing is not applicable to this article as no new data were created or analyzed in this study.
