# Supplementary material for: Subclinical Myocardial Dysfunction Assessment Using Speckle Tracking Echocardiography in Patients With Psoriasis: A Pilot Meta‐Analysis
Source: Clin Cardiol. 2024 Dec 11;47(12):e70047. doi: 10.1002/clc.70047 (PMC11632626; doi:10.1002/clc.70047)
Supplement: Supplementary file 1 — Supporting information. [file CLC-47-e70047-s001.docx]

**Supplementary Material**

**Subclinical Myocardial Dysfunction Assessment Using Speckle Tracking Echocardiography in Patients with Psoriasis: A Pilot Meta-Analysis**

**Supplementary Table S1**. The Preferred Reporting Items for Systematic Reviews and Meta-Analyses (PRISMA) 2020 Checklist.

**Supplementary Table S2**. Search strategy for all databases.

**Supplementary Table S3:** Inclusion and exclusion criteria in each study.

**Supplementary Table S4**. Quality assessment using Newcastle Ottawa Scale.

**Supplementary Figure S1**. Funnel plot for global longitudinal strain.

**Supplementary Figure S2**. Funnel plot for global circumferential strain.

The authors have provided this supplemental material to give readers additional information about their work.

**Supplementary Table S1**. The Preferred Reporting Items for Systematic Reviews and Meta-Analyses (PRISMA) 2020 Checklist.

| **Section and Topic** | **Item #** | **Checklist item** | **Location where item is reported** |
| --- | --- | --- | --- |
| **TITLE** | | |  |
| Title | 1 | Identify the report as a systematic review. | 1 |
| **ABSTRACT** | | |  |
| Abstract | 2 | See the PRISMA 2020 for Abstracts checklist. | 1,2 |
| **INTRODUCTION** | | |  |
| Rationale | 3 | Describe the rationale for the review in the context of existing knowledge. | 3 |
| Objectives | 4 | Provide an explicit statement of the objective(s) or question(s) the review addresses. | 3 |
| **METHODS** | | |  |
| Eligibility criteria | 5 | Specify the inclusion and exclusion criteria for the review and how studies were grouped for the syntheses. | 4, 5 |
| Information sources | 6 | Specify all databases, registers, websites, organisations, reference lists and other sources searched or consulted to identify studies. Specify the date when each source was last searched or consulted. | 4, 5 |
| Search strategy | 7 | Present the full search strategies for all databases, registers, and websites, including any filters and limits used. | 4, 5 |
| Selection process | 8 | Specify the methods used to decide whether a study met the inclusion criteria of the review, including how many reviewers screened each record and each report retrieved, whether they worked independently, and if applicable, details of automation tools used in the process. | 4, 5 |
| Data collection process | 9 | Specify the methods used to collect data from reports, including how many reviewers collected data from each report, whether they worked independently, any processes for obtaining or confirming data from study investigators, and if applicable, details of automation tools used in the process. | 4, 5 |
| Data items | 10a | List and define all outcomes for which data were sought. Specify whether all results that were compatible with each outcome domain in each study were sought (e.g. for all measures, time points, analyses), and if not, the methods used to decide which results to collect. | 4, 5 |
|  | 10b | List and define all other variables for which data were sought (e.g. participant and intervention characteristics, funding sources). Describe any assumptions made about any missing or unclear information. | 4, 5 |
| Study risk of bias assessment | 11 | Specify the methods used to assess risk of bias in the included studies, including details of the tool(s) used, how many reviewers assessed each study and whether they worked independently, and if applicable, details of automation tools used in the process. | 4, 5 |
| Effect measures | 12 | Specify for each outcome the effect measure(s) (e.g. risk ratio, mean difference) used in the synthesis or presentation of results. | 4, 5 |
| Synthesis methods | 13a | Describe the processes used to decide which studies were eligible for each synthesis (e.g. tabulating the study intervention characteristics and comparing against the planned groups for each synthesis (item #5)). | 5 |
|  | 13b | Describe any methods required to prepare the data for presentation or synthesis, such as handling of missing summary statistics, or data conversions. | 5 |
|  | 13c | Describe any methods used to tabulate or visually display results of individual studies and syntheses. | 5 |
|  | 13d | Describe any methods used to synthesize results and provide a rationale for the choice(s). If meta-analysis was performed, describe the model(s), method(s) to identify the presence and extent of statistical heterogeneity, and software package(s) used. | 5 |
|  | 13e | Describe any methods used to explore possible causes of heterogeneity among study results (e.g. subgroup analysis, meta-regression). | 5 |
|  | 13f | Describe any sensitivity analyses conducted to assess robustness of the synthesized results. | 5 |
| Reporting bias assessment | 14 | Describe any methods used to assess risk of bias due to missing results in a synthesis (arising from reporting biases). | NR |
| Certainty assessment | 15 | Describe any methods used to assess certainty (or confidence) in the body of evidence for an outcome. | NR |
| **RESULTS** | | |  |
| Study selection | 16a | Describe the results of the search and selection process, from the number of records identified in the search to the number of studies included in the review, ideally using a flow diagram. | 6 |
|  | 16b | Cite studies that might appear to meet the inclusion criteria, but which were excluded, and explain why they were excluded. | 6 |
| Study characteristics | 17 | Cite each included study and present its characteristics. | 6 |
| Risk of bias in studies | 18 | Present assessments of risk of bias for each included study. | 6 |
| Results of individual studies | 19 | For all outcomes, present, for each study: (a) summary statistics for each group (where appropriate) and (b) an effect estimate and its precision (e.g. confidence/credible interval), ideally using structured tables or plots. | 6, 7 |
| Results of syntheses | 20a | For each synthesis, briefly summarise the characteristics and risk of bias among contributing studies. | 6, 7 |
|  | 20b | Present results of all statistical syntheses conducted. If meta-analysis was done, present for each the summary estimate and its precision (e.g. confidence/credible interval) and measures of statistical heterogeneity. If comparing groups, describe the direction of the effect. | 6, 7 |
|  | 20c | Present results of all investigations of possible causes of heterogeneity among study results. | 6, 7 |
|  | 20d | Present results of all sensitivity analyses conducted to assess the robustness of the synthesized results. | 6, 7 |
| Reporting biases | 21 | Present assessments of risk of bias due to missing results (arising from reporting biases) for each synthesis assessed. | NR |
| Certainty of evidence | 22 | Present assessments of certainty (or confidence) in the body of evidence for each outcome assessed. | NR |
| **DISCUSSION** | | |  |
| Discussion | 23a | Provide a general interpretation of the results in the context of other evidence. | 7,8 |
|  | 23b | Discuss any limitations of the evidence included in the review. | 10 |
|  | 23c | Discuss any limitations of the review processes used. | 10 |
|  | 23d | Discuss implications of the results for practice, policy, and future research. | 9,10 |
| **OTHER INFORMATION** | | |  |
| Registration and protocol | 24a | Provide registration information for the review, including register name and registration number, or state that the review was not registered. | 4 |
|  | 24b | Indicate where the review protocol can be accessed, or state that a protocol was not prepared. | 4 |
|  | 24c | Describe and explain any amendments to information provided at registration or in the protocol. | 4 |
| Support | 25 | Describe sources of financial or non-financial support for the review, and the role of the funders or sponsors in the review. | Title Page |
| Competing interests | 26 | Declare any competing interests of review authors. | Title Page |
| Availability of data, code and other materials | 27 | Report which of the following are publicly available and where they can be found: template data collection forms; data extracted from included studies; data used for all analyses; analytic code; any other materials used in the review. | Title Page |

**Supplementary Table S2**. Search strategy for all databases.

| **No.** | **Database** | **Search Strategy** | **Number of Articles** |
| --- | --- | --- | --- |
| 1. | PubMed | (psoriasis) AND ((echocardiography) OR (two-dimensional speckle tracking) OR (strain)) | 430 |
| 2. | Embase | (psoriasis) AND ((echocardiography) OR (two-dimensional speckle tracking) OR (strain)) | 346 |
| 3. | Google Scholar | (psoriasis) AND ((echocardiography) OR (two-dimensional speckle tracking) OR (strain)) | 698 |
| 4. | Scopus | (TITLE-ABS-KEY(psoriasis)) AND (TITLE-ABS-KEY(two-dimensional speckle tracking) OR TITLE-ABS-KEY(echocardiography) OR TITLE-ABS-KEY(strain)) | 341 |
| 5. | Cochrane | (MeSH descriptor: [psoriasis] explode all trees) AND ((MeSH descriptor: [echocardiography] explode all trees) OR (“two-dimensional speckle tracking” OR “strain”)) | 12 |
| TOTAL | | | 1827 |

**Supplementary Table S3:** Inclusion and exclusion criteria in each study.

| **Author (Year)** | **Inclusion Criteria** | **Exclusion Criteria** |
| --- | --- | --- |
| Cevik 2019 | The study group was recruited from the patients under follow-up with a diagnosis of pediatric psoriasis in outpatient clinics of the Marmara University Dermatology Department. The control group consisted of healthy children. | Children with other risk factors such as obesity, diabetes, and renal or liver disease and those who were taking oral medications that may affect the cardiovascular system were excluded from the study. Patients for whom the echocardiographic image quality was poor were also excluded. |
| Dattilo 2017 | Patients were enrolled from the Department of Dermatology where they attended for periodic follow-up. Only patients with mild psoriasis with no CV risk factors and no systemic drug therapy were enrolled. The control group consisted of healthy volunteers enrolled between the staff members of both the Departments of Cardiology and Dermatology . | Excluded participants with (1) PASI 10, (2) any drug therapy, (3) smoking, (4) hypertension, (5) diabetes mellitus, (6) renal failure, (7) LV kinetic abnormalities due to left bundle branch block and/or pacemaker stimulation, (8) LV ejection fraction <50%, (9) chronic CAD, (10) arrhythmias, (11) previous cardiac surgery, (12) cardiomyopathies, (13) moderate-to-severe valve disorders, and (14) poor acoustic window. |
| Duman 2019 | Forty patients who were over the age of 18 years, had been diagnosed with psoriasis vulgaris for a minimum of 3 years via biopsy, and admitted to the Dermatology Department, Faculty of Medicine, Recep Tayyip Erdoğan University, Rize, Turkey (group 1), were selected as study participants. Additionally, 40 age- and sex-matched healthy individuals were included in this study as the control group (group 2). | Patients and control subjects with a history of past or concurrent diseases such as hypertension (HT), diabetes mellitus, coronary artery disease (CAD), lung diseases and/or pulmonary HT, valvular heart diseases, liver or kidney diseases, collagen vascular diseases, rhythms other than sinus, any cardiovascular drug use, abnormal thyroid function, or serum electrolyte values were excluded from the study. Also, obese subjects (BMI ≥ 30 kg/m2) were excluded from the study. Patients with coexisting psoriatic arthritis (diagnosed by a rheumatologist) were excluded due to the possible adverse effects on heart rhythm. |
| Gullo 2018 | To be selected for the study, subjects needed to fulfill the following inclusion criteria: (i) to be newly diagnosed, (ii) not being exposed to immunomodulatory treatments, (iii) to be free of traditional CV risk factors, and (iv) to meet the classification criteria for PsA or RA. Traditional CV risk factors were screened according to national guidelines: diabetes mellitus, dyslipidemia (total-cholesterol ≥ 230 mg/dl or low-density lipoprotein cholesterol (LDL-C)≥160 mg/dl, or triglycerides ≥ 250 mg/dl), hypertension (systolic blood pressure (SBP) ≥140 mmHg and/or diastolic blood pressure (DBP) ≥ 90 mmHg). | Exclusion criteria were: treatment with any immunosuppressive drugs, long-term corticosteroids, NSAIDs or DMARDs, smoking habit, usage of contraceptive/hormone-based therapies, presence of thyroid, liver or kidney diseases (previous diagnosis of kidney disease or decreased glomerular filtration rate <60ml/min/1.73m2), body mass index (BMI)>30, alcohol consumption, previous history of CVD and abnormal electrocardiographic or echocardiographic (left ventricular ejection function, left ventricular regional function) pattern |
| Ikonomidis 2015 | We studied 59 patients with psoriasis matched by age and sex with 59 patients with angiographically documented CAD ( 70% luminal diameter stenosis) and preserved ejection fraction (EF) > 50%.  Forty participants with age, sex, and atherosclerotic risk factors similar to those of the patients with psoriasis rwith normal electrocardiograms, echocardiograms, treadmill test results, and stress echocardiogramsdwere selected as a control group with low risk for obstructive CAD from participants attending the cardiology outpatient clinic.  All patients with psoriasis received treatment with cyclosporine 2.5-3 mg/kg daily. The disease duration from initial diagnosis until inclusion in the study was 61.3 ± 15 months. All patients had plaque-type psoriasis, and no one had psoriatic arthritis or inflammatory bowel syndrome.  Regarding the group of patients with CAD, we included 59 patients who fulfilled all the following criteria: (1) exercise and stress-related angina, (2) evidence of reversible ischemia during a treadmill exercise test and stress echocardiography, and (3) angiographically documented stenosis of 70% in 1 or several of the major coronary arteries within 1 year before inclusion in the study. | Exclusion criteria for patients with psoriasis were presence of wall motion abnormalities and EF ≤ 50%, psoriatic arthritis, history of acute coronary syndrome, familial hyperlipidemia, diabetes mellitus, chronic obstructive pulmonary disease or asthma, moderate or severe valvular heart disease, primary cardiomyopathies, and malignant tumours. CAD was excluded in patients with psoriasis by the absence of clinical history, angina, and reversible myocardial ischemia as assessed by a treadmill test and stress echocardiography.  Exclusion criteria were a history of ST- elevation myocardial infarction, presence of wall motion ab- normalities or EF of ≤ 50%, or both, to exclude the presence of transmural scar compromising myocardial deformation indices. Other exclusion criteria were history of acute coronary syndrome without ST-segment elevation within the past year, familial hyperlipidemia, diabetes mellitus, chronic obstructive pulmonary disease or asthma, moderate or severe valvular heart disease, and malignant tumours. |
| Karabay 2019 | The study group comprised 30 patients with untreated psoriasis, no risk factors for CVD, and no systemic disease, as confirmed by a physical examination, laboratory tests, and imaging studies. The subjects were recruited from the Department of Dermatology. For comparison, 20 healthy controls without psoriasis were enrolled from among hospital staff volunteers. Only those with normal BMI (18.5-25 kg/m2) were included. | Subjects with a history of topical or systemic psoriasis therapy were excluded. Subjects were excluded if they had a documented history of CVD, smoking habit and/or alcohol consumption. BMI and conventional risk factors for CVD, such as history of smoking, diabetes mellitus, hypercholesterolemia, and hypertension were excluded. |
| Pletikosic 2023 | Twenty-five healthy Caucasian controls, ages 18–65 years, without cardiovascular disease were randomly selected . | Exclusion criteria was the presence of arterial hypertension, coronary heart disease, valvular heart disease, chronic heart failure, history of transient ischemic attack or cerebrovascular insult, diabetes mellitus, moderate or severe chronic kidney disease defined as estimated glomerular filtration rate (eGFR)<60 ml/min, aortic/peripheral arterial disease, clinically significant arrhythmia, smoking or alcohol use (≥ 3 drinks per week). |
| Sen 2016 | Fourty consecutive patients diagnosed with psoriasis vulgaris by clinical or histopathological examination were included. The control subjects (n = 35) had normal physical examination, chest roentgenogram, electrocardiogram, and 2-D and Doppler echocardiogram findings, and none of them had CV or any other organ system disease. | Subjects with hypertension, renal failure, diabetes mellitus, LV EF of <50%, moderate or severe valvular stenosis and/or regurgitation, coronary artery disease, chronic obstructive pulmonary disease, or atrial fibrillation were excluded. Subjects in whom echocardiographic image quality was poor were also excluded. |
| Shang 2014 | One hundred five patients were consecutively screened at the rheumatology clinic of Prince of Wales Hospital affiliated to the Chinese University of Hong Kong from January 2007 to May 2008. Seventy-six patients, who were aged > 18 years and who fulfilled the Classification of Psoriatic Arthritis criteria23, were recruited for this study, including 33 patients without traditional CV risk factors. Twenty-four healthy control subjects without a history of overt CVD were recruited from the community through advertisement or from other clinics. | Exclusion criteria included pregnancy, hypothyroidism, clinically significant renal disease (serum creatinine level ≥ 270 μmol/l), history of angina, stable coronary artery disease, previous acute coronary syndromes, coronary revascular- ization, bundle-branch block, second-degree or higher atrioventricular block, atrial fibrillation, valvular stenosis or at least moderate valvular regurgitation, valvular replacement or repair, or mitral annular calcification. |
| Skokr 2019 | The study included patients at any age, with psoriasis who do not have chronic or autoimmune diseases and are able to understand instructions and provide informed consent. | Known hypertensive, diabetic patients, documented ischemic heart disease, patients with any type of pulmonary hypertension, patients with congenital heart disease, atrial fibrillation (AF), patients with reduced EF ≤ 50%, poor image quality on echocardiographic window, significant comorbidities, patients with bad compliance, uncooperative patients, patients that refused the consent or the study or inability to give informed consent were excluded from the study. |
| Zhao 2014 | From January 2007 to January 2010, consecutive Chinese patients aged between 18 and 55 years old with chronic plaque psoriasis that involved >10% of the body surface area were recruited from the Dermatology outpatient clinic of Queen Mary Hospital, Hong Kong. During the study period, 53 age- and gender-matched Chinese controls with no evidence of psoriasis or other systemic inflammatory disease were recruited for comparison from a community health screening program. | Patients with clinical or radiological evidence of psoriatic arthritis were carefully excluded to eliminate the potential confounding effect of systemic arthritis. Patients were also excluded if they had a documented history of cardiovascular disease including coronary artery disease, myocardial infarction, stroke or peripheral vascular disease, significant renal impairment with creatinine >220 µmol/L, liver failure or clinical/biochemical evidence of concomitant inflammatory disease. Screening of 94 patients with psoriasis revealed that eight had a documented history of cardiovascular disease and 12 refused to participate in the study. |

**Supplementary Table S4**. Quality assessment using Newcastle Ottawa Scale.

| **Study**  **(Year)** | **Selection** | | | | **Comparability** | **Outcome** | | | **Total Score** |
| --- | --- | --- | --- | --- | --- | --- | --- | --- | --- |
|  | Representativeness of the exposed cohort | Selection of the non-exposed cohort | Ascertainment of exposure | Outcome of interest was not present at the start of the study |  | Assessment of outcome | Follow-up duration | Adequacy of follow-up |  |
| Sen 2016 | ☆ | ☆ | ☆ | ☆ | ☆☆ | ☆ | ☆ |  | 8 |
| Karabay 2019 | ☆ | ☆ |  | ☆ | ☆ | ☆☆ | ☆ | ☆ | 8 |
| Gullo 2018 | ☆ | ☆ | ☆ | ☆ | ☆☆ | ☆☆ | ☆ |  | 9 |
| Pletikosic 2023 | ☆ | ☆ | ☆ | ☆ | ☆ | ☆ |  | ☆ | 7 |
| Ikonomidis 2015 | ☆ | ☆ | ☆ | ☆ | ☆ | ☆☆ | ☆ |  | 7 |
| Cevik 2019 | ☆ | ☆ | ☆ |  | ☆☆ | ☆ |  | ☆ | 7 |
| Zhao 2014 | ☆ | ☆ |  | ☆ | ☆ | ☆☆ | ☆ | ☆ | 8 |
| Skokr 2019 | ☆ | ☆ | ☆ | ☆ | ☆ | ☆ |  | ☆ | 7 |
| Dattilo 2017 | ☆ | ☆ |  |  | ☆☆ | ☆☆ | ☆ | ☆ | 8 |
| Duman 2019 | ☆ | ☆ | ☆ | ☆ | ☆ | ☆ | ☆ |  | 7 |
| Shang 2014 | ☆ | ☆ |  | ☆ | ☆ | ☆ | ☆ | ☆ | 7 |

**Supplementary Figure S1**. Funnel plot for global longitudinal strain.

**
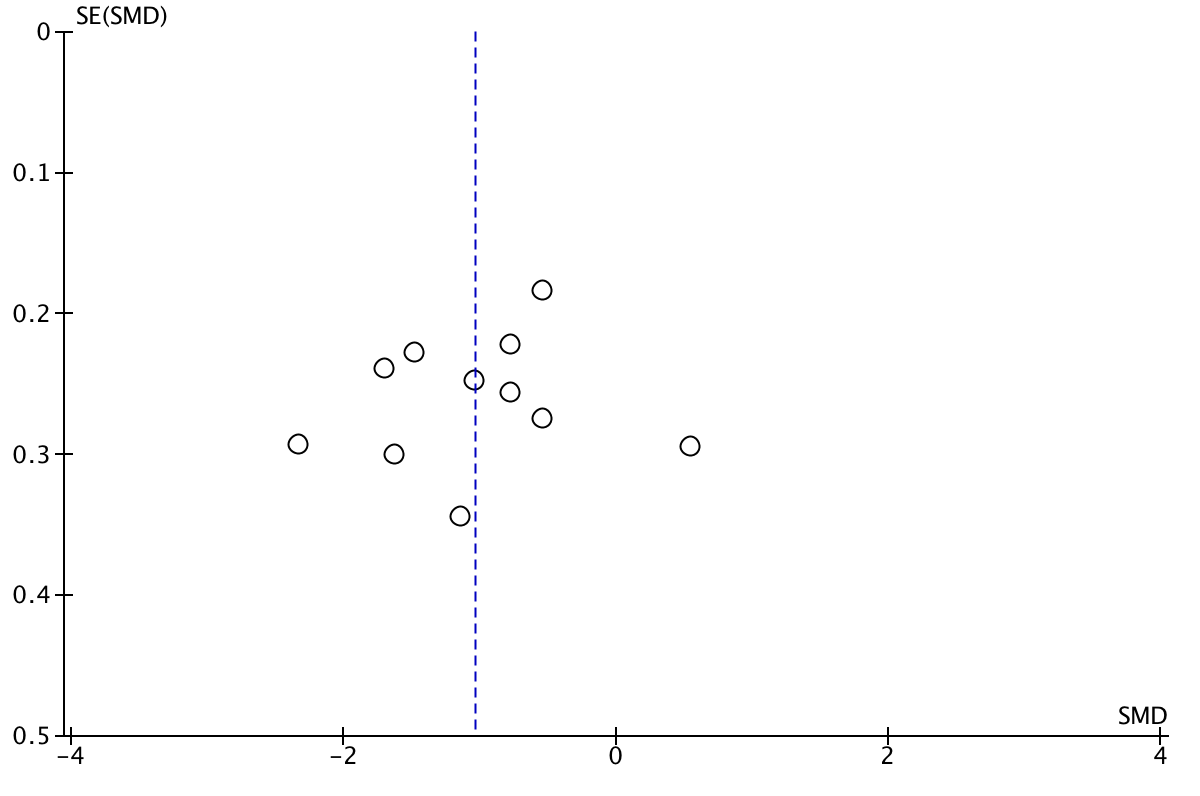
**

**Supplementary Figure S2**. Funnel plot for global circumferential strain.

**
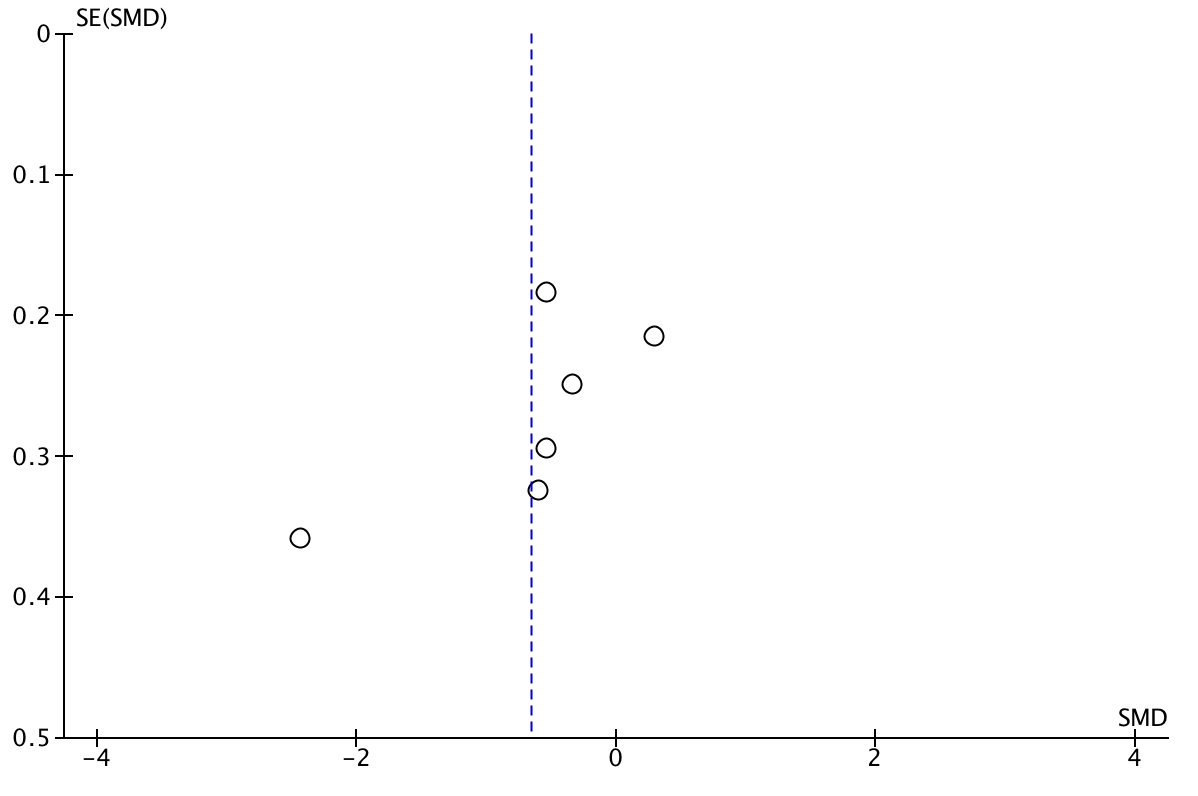
**
